# Supplementary material for: Red Blood Cell Omega-6 Fatty Acids and Biomarkers of Inflammation in the Framingham Offspring Study
Source: Nutrients. 2025 Jun 22;17(13):2076. doi: 10.3390/nu17132076 (PMC12251348; doi:10.3390/nu17132076)
Supplement: Supplementary file 1 [file nutrients-17-02076-s001.zip › nutrients-3689257-supplementary.pdf]

Table S1. Descriptive statistics for linoleic acid, arachidonic acid, and omega-3 index

Exposure (% total fatty acids)	Mean ± SD	Min	10th Percentile	Median	90th Percentile	Max
Linoleic Acid	11.04 ± 1.71	5.64	9.00	10.95	13.23	19.58
Arachidonic Acid	16.57 ± 1.60	8.87	14.49	16.68	18.48	21.17
Omega-3 Index	5.57 ± 1.71	1.81	3.60	5.29	7.86	15.12

Table S2. Partial correlations between the omega-3 index and 10 inflammatory biomarkers with and without adjustment for RBC linoleic acid and arachidonic acid (All biomarkers log-transformed and standardized)

Inflammatory Biomarker	Model 1	Model 2
Isoprostanes/Creatinine	-0.137 (-0.174, -0.101)**	-0.115 (-0.152, -0.077)**
CRP	-0.131 (-0.168, -0.094)**	-0.069 (-0.104, -0.034)**
Interleukin-6	-0.142 (-0.178, -0.107)**	-0.101 (-0.136, -0.066)**
ICAM-1	-0.118 (-0.155, -0.081)**	-0.062 (-0.098, -0.026)**
LpPLA2 Activity	-0.087 (-0.123, -0.051)**	-0.048 (-0.079, -0.017)**
LpPLA2 Mass	-0.133 (-0.171, -0.096)**	-0.078 (-0.114, -0.042)**
MCP-1	-0.070 (-0.106, -0.033)**	-0.038 (-0.075, 0.000)
Osteoprotegerin	-0.068 (-0.101, -0.034)**	-0.052 (-0.086, -0.018)**
P-selectin	-0.102 (-0.140, -0.065)**	-0.068 (-0.105, -0.030)**
TNFR2	-0.085 (-0.121, -0.050)**	-0.060 (-0.096, -0.024)**

CRP = C-reactive protein; ICAM-1 = intercellular adhesion molecule-1; Lp-PLA2 = Lipoprotein-associated phospholipase-A2; MCP-1 = monocyte chemattractant protein-1; TNFR2 = tumor necrosis factor receptor-2
**p<0.01.
Model 1: Age and Sex
Model 2: all variables in Table 2 plus LA% and AA%

Table S3. The association between arachidonic acid and osteoprotegerin by quintiles

Quintile	Median AA (% of Total FA)	Median Osteoprotegerin (Log-Transformed)	N	Beta	SE	P-Value
Q1 (ref)	14.48	1.57	555	0	0	0
Q2	15.88	1.55	553	-0.045	0.05	0.391
Q3	16.67	1.58	557	0.016	0.05	0.761
Q4	17.43	1.51	556	-0.164	0.05	0.002
Q5	18.48	1.56	556	-0.108	0.05	0.046

Model adjusted for all Table 2 variables (except FAs)

Table S4. The association between arachidonic acid and intercellular adhesion molecule 1 stratified by ethnicity

Group	Median AA (% of Total FA)	Median ICAM-1 (Log-Transformed)	N	Beta	SE	P-Value
Non-Hispanic Whites	16.67	5.62	2490	-0.105	0.03	<0.001
Non-White and/or Hispanic	16.72	5.45	266	-0.589	0.14	<0.001

Model adjusted for all Table 2 variables + LA + O3I
